# Beyond the Standard Antibiogram: Unveiling the Roles of pH and Salinity in Modulating Gentamicin Efficacy Against *Escherichia coli* in Urinary Tract Infections

**DOI:** 10.1002/mbo3.70375

**Published:** 2026-08-13

**Authors:** Abdelwahab Rai, Mahdia El‐djohra El‐amina Saoud, Nesrine Tabchouche, Hamdi Bendif, Stefania Garzoli, Lotfi Mouni

**Affiliations:** ^1^ Laboratoire de Gestion et Valorisation des Ressources Naturelles et Assurance Qualité, MicroSOS, Faculté SNVST Université de Bouira Bouira Algeria; ^2^ Department of Biology, College of Science Imam Mohammad Ibn Saud Islamic University (IMSIU) Riyadh Saudi Arabia; ^3^ Department of Chemistry and Technologies of Drug Sapienza University Rome Italy

**Keywords:** antimicrobial resistance, in vitro infection modeling, osmoregulation, phenotypic plasticity, therapeutic failure

## Abstract

Urinary tract infections are highly prevalent diseases with a broad spectrum of clinical manifestations. This study investigates the influence of two key abiotic factors, pH and salinity, on the growth and antibiotic susceptibility of *Escherichia coli* ATCC 25922, *Pseudomonas aeruginosa* ATCC 27853, and a clinical *E. coli* isolate obtained from a human urine sample. Growth kinetics and gentamicin susceptibility were evaluated across a pH range (4.5–11.0) and NaCl concentrations (0–1000 mM) using Luria–Bertani and Mueller–Hinton (MH) media. In parallel, real urine was analyzed, and urine‐mimicking MH media were prepared by adjusting pH and salinity independently and in combination. Gentamicin susceptibility was strongly influenced by environmental conditions. Salinity pre‐exposure increased *E*. *coli* and *P*. *aeruginosa* resistance from 23.0 to 19.0 mm and from 25.5 to 18.5 mm at 800 mM, respectively. Direct pH modification enhanced gentamicin activity at extreme pH (up to 34.33 mm in *E. coli* and 33.5 mm in *P. aeruginosa*). In standard MH medium, the clinical *E*. *coli* isolate exhibited a 32.5‐mm inhibition zone and a gentamicin minimum inhibitory concentration (MIC) of 4 µg/mL. Under urine‐mimicking conditions, the inhibition zone decreased to 22.5 mm, while the MIC increased eightfold to 32 µg/mL, indicating a marked reduction in gentamicin efficacy under physiologically relevant urinary conditions. These findings demonstrate that environmental stress profoundly influences gentamicin efficacy and suggest that this effect may involve physiological adaptations, including RpoS‐mediated stress responses, membrane remodeling, and osmoprotective mechanisms previously described in the literature.

## Graphical abstract

Modulation of the efficacy of gentamicin in urinary tract infections.

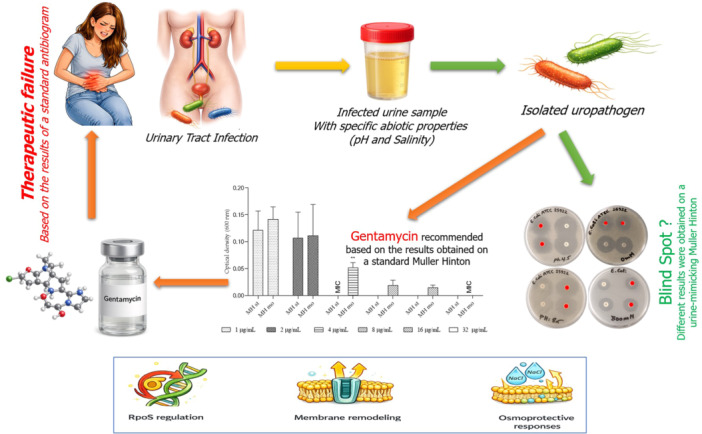

## 1 Introduction

Urinary tract infections (UTIs) are among the most common bacterial infections worldwide and represent a major public health burden, particularly affecting women, the elderly, and immunocompromised individuals (Saenz et al. 2024). They arise from microbial colonization of the urinary tract and present diverse clinical manifestations depending on infection site, pathogen, and host immune status (Griebling 2007).

Among uropathogens, *Escherichia coli* predominates, accounting for over 80% of community‐acquired UTIs. Other contributors include *Staphylococcus saprophyticus*, *Klebsiella* spp., *Proteus mirabilis*, and *Enterococcus faecalis* (Sokhn et al. 2020; Bidet et al. 2012; Hackel et al. 2025). *E. coli* functions both as a dominant intestinal commensal and as a versatile pathogen capable of adapting to extraintestinal environments, such as the urinary tract (Smati et al. 2015; Y. Zhang et al. 2025).

Antimicrobial susceptibility testing (AST) via standardized antibiograms guides empirical therapy (Joshi 2010; Abejez‐Arrizabalaga et al. 2025). However, results depend heavily on laboratory conditions, including the physicochemical properties of the test medium. Variations in pH and ionic strength can alter apparent susceptibility, potentially creating discrepancies between in vitro findings and clinical outcomes (Weber 2003; Truong et al. 2021; Simner et al. 2022). Moreover, standard antibiograms primarily assess growth inhibition and do not account for antibiotic interactions or infection‐site environmental conditions (Henniche et al. 2021; Kmiecikowski et al. 2025; Fair and Tor 2014; Meyer et al. 2016).

Urine represents a dynamic biochemical environment characterized by variable acidity and salinity. Such conditions can influence bacterial physiology and antibiotic pharmacodynamics (Fair and Tor 2014; Coronado et al. 1995). Gentamicin, a widely used aminoglycoside for severe Gram‐negative infections, exhibits activity that is strongly environment‐dependent (Buxeraud and Faure 2016; Dagur et al. 2023). Its bactericidal effect relies on membrane potential–driven uptake and ribosomal binding (Shiraliyev and Orman 2024), while acidic pH or elevated salinity may reduce efficacy through structural instability or altered membrane permeability (Coronado et al. 1995; Wiegand et al. 2015).

Bacterial responses to osmotic and acid stress involve membrane remodeling, altered protein expression, and activation of global regulators, such as RpoS (Y. J. Zhang et al. 2019; Battesti et al. 2011; Dong and Schellhorn 2010; Valencia et al. 2024). In *E. coli*, osmoprotective mechanisms, including K^+^ uptake and betaine accumulation via the ProU system, support adaptation under high salinity (Lucht and Bremer 1994; Kloss et al. 2025; Thomas et al. 2025), whereas acid stress disrupts membrane integrity and intracellular homeostasis (Baatout et al. 2007; Bearson et al. 1997; Li et al. 2024; Guan and Liu 2020). These stress responses may ultimately modify antibiotic uptake and susceptibility (Abraham and Duthie 1946; Aranda‐Díaz et al. 2020).

Despite the widespread use of standardized AST, conventional Mueller–Hinton (MH) media do not reproduce the physicochemical conditions encountered at the site of infection, particularly in the urinary tract. Consequently, the influence of urine‐mimicking conditions on gentamicin susceptibility remains insufficiently explored. To address this gap, we investigated how variations in pH and salinity influence gentamicin efficacy. The initial experiments employed a broad range of pH and NaCl concentrations to investigate the physiological limits of bacterial adaptation and their impact on gentamicin activity against reference strains. Clinical relevance was subsequently assessed using MH media adjusted to the physicochemical properties of a real urine sample, allowing the evaluation of gentamicin susceptibility against a clinical *E*. *coli* isolate under urine‐mimicking conditions. By incorporating physiologically relevant urinary conditions into susceptibility testing, we aim to improve the clinical relevance of antibiogram interpretation. *Pseudomonas aeruginosa* ATCC 27853 was included in this investigation as a well‐established quality‐control strain for AST and a clinically relevant opportunistic uropathogen associated with complicated UTIs.

## 2 Material and Methods

The work was realized in two separated parts. In the initial phase of the study, we investigated how variations in pH and salinity (NaCl concentration) in MH medium affect the in vitro efficacy of gentamicin against two reference bacterial strains: *E. coli* ATCC 25922 and *P. aeruginosa* ATCC 27853. They were selected because they are recognized as quality‐control reference strains with well‐characterized antimicrobial susceptibility profiles, ensuring reproducibility and comparability of the experimental results. In the second part, a clinical *E. coli* isolate obtained from a urine sample was subjected to various antibiograms under urine‐simulating pH and salinity (electrical conductivity, EC) conditions. The isolate was identified using standard clinical microbiology procedures, as described below. This part of the study aimed to evaluate the interaction between bacteria and gentamicin under varying physicochemical conditions in the urinary environment.

### 2.1 Effect of pH and Salinity Variations on Bacterial Growth

This experiment was conducted to evaluate the growth responses of *E. coli* ATCC 25922 and *P. aeruginosa* ATCC 27853 under varying pH and salinity conditions in a complex nutrient medium. The objective was to assess the tolerance and adaptability of both strains to environmental stressors, specifically alterations in hydrogen‐ion concentration (pH) and sodium chloride (NaCl) concentration.

#### 2.1.1 Salinity Stress Assay

The halotolerance of *E. coli* ATCC 25922 and *P. aeruginosa* ATCC 27853 was assessed using Luria–Bertani (LB) broth as a nutrient‐rich culture medium. The experiment began with a control condition using standard LB medium without additional NaCl to establish baseline growth parameters under nonsaline conditions. Subsequent test conditions included LB media supplemented with increasing concentrations of sodium chloride: 200, 400, 800, and 1000 mM, to simulate incremental salinity stress.

For each condition, 5 mL of the respective LB medium was aliquoted into sterile, sealed culture tubes. Each tube was inoculated with 200 µL of a bacterial suspension adjusted to 0.5 McFarland standard, ensuring uniform initial cell density across all samples. Inoculated tubes were incubated statically at 37°C for 24 h.

Following incubation, bacterial growth was quantitatively evaluated by measuring the optical density at 620 nm (OD_620_) using an OPTIZEN spectrophotometer. Growth measurements were used to assess the tolerance and adaptability of each bacterial strain across the salinity gradient.

#### 2.1.2 pH Stress Assay

To evaluate the influence of pH on bacterial growth, LB broth was adjusted to five pH values representing a broad physiological range: 4.5, 6.0, 7.3 (neutral), 8.5, and 11.0. The pH adjustments were made using either HCl or NaOH, and the media were sterilized prior to inoculation.

For each condition, 5 mL of the pH‐adjusted LB medium was dispensed into sterile, sealed culture tubes. Each tube was inoculated with 200 µL of a bacterial suspension standardized to 0.5 McFarland turbidity to ensure a consistent initial bacterial density. All cultures were incubated statically at 37°C for 24 h.

Post‐incubation, bacterial growth was assessed by measuring OD_620_ using an OPTIZEN spectrophotometer. The resulting data were used to determine the growth profiles of the bacterial strains across the tested pH range.

### 2.2 Antibiograms Under Standard and Modified Conditions

AST was performed to assess the impact of environmental stressors, specifically salinity and pH, on the activity of gentamicin against *E. coli* ATCC 25922 and *P. aeruginosa* ATCC 27853. Two experimental approaches were employed. In the first, bacterial cultures were pre‐exposed to salinity or pH stress prior to antibiotic testing. In the second, the stressor was incorporated directly into the MH agar medium used for antibiogram assays.

#### 2.2.1 Antibiogram Under Salinity Stress

##### 2.2.1.1 Stress Applied to Cultures Before Antibiograms Realization

To simulate osmotic preconditioning, LB broth was prepared with varying NaCl concentrations: 0 (control), 0.2, 0.4, 0.6, and 0.8 M. For each salinity level, 5 mL of LB medium was dispensed into sterile conical tubes and inoculated with a fresh (24‐h) bacterial culture of either *E. coli* ATCC 25922 or *P. aeruginosa* ATCC 27853.

Following incubation at 37°C for 24 h under static conditions, cultures were centrifuged at 3000 rpm for 15 min. The resulting bacterial pellet was resuspended in 5 mL of sterile physiological saline. Bacterial concentration was adjusted to match a 0.5 McFarland turbidity standard (~1 × 10^8^ CFU/mL). This suspension was used for subsequent antibiotic susceptibility testing.

##### 2.2.1.2 Stress Applied Directly to the Antibiogram Media

To evaluate the direct effect of salinity on gentamicin activity, modified MH agar was prepared by dissolving 7.6 g of MH powder in 200 mL of distilled water, then adding NaCl at concentrations of 0.2, 0.4, 0.6, or 0.8 M. The media were heated under continuous stirring until fully dissolved, then sterilized by autoclaving at 121°C for 15 min. Gentamicin susceptibility testing was then conducted on these modified media.

#### 2.2.2 Antibiogram Under pH Stress

##### 2.2.2.1 Stress Applied to Cultures Before Antibiograms Realization

To simulate pH stress during bacterial growth, LB broth was adjusted to target pH values of 4.5, 6.0, 7.3, and 8.5 prior to sterilization. Each pH‐adjusted medium (5 mL) was inoculated in sterile conical tubes with a 24‐h culture of the test strains. After incubation at 37°C for 24 h, cultures were centrifuged at 3000 rpm for 15 min. The bacterial pellets were then resuspended in 5 mL of physiological saline. Cell suspensions were adjusted to a 0.5 McFarland standard and used for the antibiogram.

##### 2.2.2.2 Stress Applied Directly to the Antibiograms' Media

MH agar medium was modified by adjusting the pH of the medium prior to sterilization. Briefly, 7.6 g of MH powder was suspended in 200 mL of distilled water. The pH was adjusted to 4.5, 6.0, 7.3, or 8.5 using sterile HCl or NaOH as appropriate. The media were heated with agitation and autoclaved at 121°C for 15 min.

#### 2.2.3 Antibiogram Procedure

Following preparation of the inocula (standard and stress‐adapted), the antibiogram was performed using the standard disc diffusion (swabbing) method on MH agar plates. Sterile swabs were used to uniformly inoculate the agar surface with the adjusted bacterial suspensions. Gentamicin discs (containing the specified antibiotic concentration) were aseptically placed onto the agar surface.

Plates were incubated for 18–24 h at a controlled temperature of 37°C. Inhibition zone diameters were measured in millimeters. The procedure was conducted in accordance with the methodology described by Chardon (2008), ensuring consistency with established AST standards.

### 2.3 Simulation of Real Urine Properties

#### 2.3.1 Urine Sampling

A midstream urine sample was collected from a consenting adult donor at the Betouche Medical Analysis Laboratory in Bouira, Algeria, in accordance with hygienic protocols to minimize contamination. An initial volume of approximately 20 mL of the urinary flow was discarded, and the subsequent stream was collected into a sterile, labeled container. The sample was immediately stored at 4°C in a transport cooler.

Upon arrival at the SNVST Faculty Laboratory (University of Bouira), the sample was divided into two aliquots: one for the isolation of uropathogenic bacteria and the other for physicochemical analysis (pH and EC). Immediate processing was ensured to limit microbial proliferation.

#### 2.3.2 Urine Characterization in Terms of pH and Salinity

The physicochemical properties of the urine sample, namely, pH and salinity, were measured using a calibrated multiparameter instrument (HANNA HI 9829). The probe (HI 7609829) was rinsed with distilled water between uses and then immersed directly in the urine sample for real‐time measurement. EC served as a proxy for salinity. The recorded values were subsequently used to guide the formulation of modified MH media.

#### 2.3.3 Pathogen Isolation, Subculturing, and Conservation

Microbiological analysis of the urine sample was performed at the Betouche Medical Analysis Laboratory (Bouira, Algeria). The clinical isolate was identified as *E. coli* using standard diagnostic procedures, including CHROMagar Orientation (Alonso et al. 1996), conventional biochemical tests, and the API 20E identification system. After identification, the isolate was subcultured on nutrient agar and preserved under appropriate conditions for subsequent AST.

#### 2.3.4 Effect of MH Modification on the Bacterial Response to Antibiotics

Following characterization of the urine sample, three types of MH agar media were prepared to simulate the urinary environment:

- MH adjusted to urinary pH only.
- MH adjusted to urinary EC (salinity) only.
- MH adjusted to both urinary pH and EC.

Media pH adjustments were made using sterile 1 M HCl or NaOH solutions, and salinity was modulated by the addition of analytical‐grade NaCl. Final pH and EC values were validated using the HANNA HI 9829 multiparameter device. Each prepared medium was aliquoted into sterile, labeled flasks and autoclaved at 121°C for 20 min.

Antibiograms were then conducted using the disc diffusion method on both the standard MH medium and the modified variants. Plates were uniformly inoculated with the previously prepared bacterial suspension using sterile swabs. Gentamicin discs were placed onto the agar surface, and the plates were incubated for 18–24 h at 37°C. Inhibition zones were measured in millimeters. All procedures adhered to the method described by Chardon (2008).

#### 2.3.5 Effect of MH Modifications on Gentamicin Minimum Inhibitory Concentration (MIC)

To assess the influence of urine‐simulated conditions on antibiotic efficacy, the MIC of gentamicin was determined in standard and modified MH broths (without agar). A total of 350 mL of MH broth was prepared and divided into two equal parts:

- *Standard medium:* Adjusted to pH 7.0 ± 0.2 (control).
- *Modified medium:* Adjusted to match the urinary pH and EC.

Each medium type was distributed into 25 mL aliquots in sterile flasks and autoclaved at 120°C for 20 min.

A gentamicin working solution (100 μg/mL) was prepared by diluting 1 mL of the original stock (Gentheraxine, 40 mg/mL) in 399 mL of sterile distilled water. This solution was used to generate a series of final antibiotic concentrations: 0, 1, 2, 4, 8, 16, and 32 μg/mL. The antibiotic‐containing media were dispensed into sterile 96‐well microplates at 200 μL per well. Each well was inoculated with 20 μL of the standardized bacterial suspension. The plates were incubated at 37°C for 24 h. Bacterial growth was assessed by measuring absorbance at 600 nm using a spectrophotometer (OPTIZEN).

The MIC of gentamicin was defined as the lowest concentration of the antibiotic at which no visible growth (OD ≈ baseline) was detected.

### 2.4 Statistical Analysis

All statistical analyses and figure preparation were performed using GraphPad Prism version 9.3.1 (GraphPad Software, San Diego, CA, USA). Data are presented as mean ± standard deviation (SD), and error bars represent the SD.

For experiments involving the reference strains (*E. coli* ATCC 25922 and *P. aeruginosa* ATCC 27853), data were obtained from three independent biological experiments, each including duplicate gentamicin (GN) disc measurements (technical replicates). Experiments performed under urine‐mimicking conditions using the clinical *E. coli* isolate were conducted in two independent biological replicates. Gentamicin MIC assays were performed in four independent experiments.

Differences in gentamicin activity under varying pH and salinity conditions were analyzed using one‐way analysis of variance (ANOVA) followed by Fisher's Least Significant Difference (LSD) post hoc test. MIC data obtained under standard and modified MH broth conditions were analyzed using two‐way ANOVA followed by Fisher's LSD multiple‐comparison test to evaluate the effects of gentamicin concentration, culture medium, and their interaction. Statistical significance was defined as *p* ≤ 0.05. Levels of significance are indicated in the figures as follows: *p < 0.05, **p < 0.01, ***p < 0.001, ****p < 0.0001.

## 3 Results

### 3.1 *E*. *coli* and *P*. *aeruginosa* response to pH and Salinity variations

#### 3.1.1 Effect of Salinity

**Figure 1:**
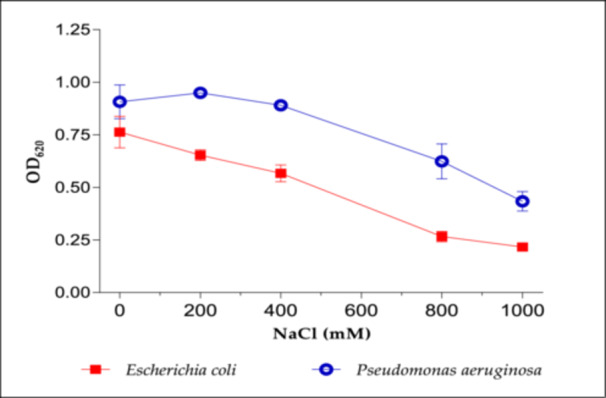
Growth response of *Escherichia coli* ATCC 25922 and *Pseudomonas aeruginosa* ATCC 27853 under varying NaCl concentrations. Bacterial cultures were incubated for 24 h in Luria–Bertani (LB) medium supplemented with 0, 200, 400, 800, and 1000 mM NaCl. Growth was measured as optical density at 620 nm (OD_620_).

The growth response of *E. coli* ATCC 25922 and *P. aeruginosa* ATCC 27853 to increasing concentrations of sodium chloride (NaCl) was assessed in LB medium. *E. coli* ATCC 25922 exhibited a progressive decline in growth with increasing salinity. Optical density (OD_600_) values decreased from 0.76 in the absence of NaCl to 0.65 and 0.56 in media supplemented with 200 and 400 mM NaCl, respectively. Notably, a sharp reduction in growth was observed at higher salt concentrations, with OD values falling to 0.26 and 0.21 at 800 and 1000 mM NaCl, respectively, indicating a marked loss in viability beyond 400 mM (Figure 1).

In contrast, *P. aeruginosa* ATCC 27853 demonstrated greater salinity tolerance under the same conditions. The strain maintained robust growth up to 400 mM NaCl, with OD values of 0.92, 0.95, and 0.89 at 0, 200, and 400 mM NaCl, respectively. However, significant growth inhibition was detected at elevated salt concentrations, with OD values declining to 0.62 and 0.43 at 800 and 1000 mM NaCl, respectively. These results indicate that *P. aeruginosa* ATCC 27853 possesses a higher threshold for salt‐induced stress compared with *E. coli* ATCC 25922 (Figure 1).

#### 3.1.2 Effect of pH

**Figure 2:**
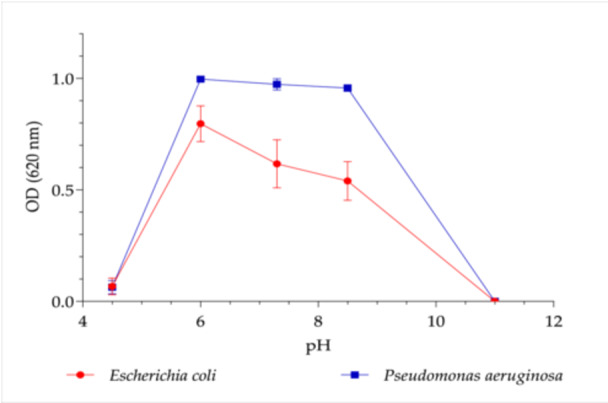
Growth response of *Escherichia coli* ATCC 25922 and *Pseudomonas aeruginosa* ATCC 27853 under varying pH conditions. Bacterial cultures were incubated for 24 h in Luria–Bertani (LB) medium adjusted to pH values of 4.5, 6.0, 7.3, 8.5, and 11.0. Growth was measured as optical density at 620 nm (OD_620_).

The influence of pH on the growth of *E. coli* ATCC 25922 and *P. aeruginosa* ATCC 27853 was evaluated in LB medium over a broad pH range. The results revealed distinct pH tolerance profiles between the two strains. *E. coli* ATCC 25922 exhibited a narrow optimal pH range, with peak growth observed at pH 6.0, corresponding to an OD_600_ of 0.79 (Figure 2). Growth decreased substantially at more alkaline conditions, with OD values declining to 0.61 at pH 7.3 and 0.54 at pH 8.5. No detectable growth was observed at pH 4.5 or pH 11, indicating a limited tolerance to both acidic and highly alkaline environments.

In contrast, *P. aeruginosa* ATCC 27853 demonstrated a broader pH tolerance. The strain maintained relatively stable and optimal growth across the pH range of 6.0–8.5, with only a significant reduction in OD observed beyond this range (Figure 2). Similar to *E. coli*, the strain did not grow at either pH 4.5 or 11.0. These results indicate that *P. aeruginosa* ATCC 27853 has a greater capacity to adapt to pH fluctuations than *E. coli* ATCC 25922.

### 3.2 Antibiogram Under Standard and Modified Conditions

#### 3.2.1 Case of *E. coli*

##### 3.2.1.1 Antibiogram Under Salinity Stress

**Figure 3:**
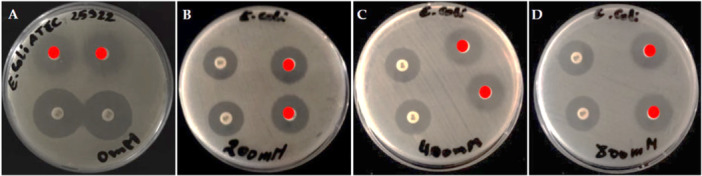
Gentamicin inhibitory activity (GN IA) against *Escherichia coli* ATCC 25922 after pre‐exposure to salinity stress in Luria–Bertani (LB) broth, prior to antibiogram testing on standard Mueller–Hinton agar medium. Cultures were grown in LB with varying NaCl concentrations for 24 h at 37°C, then processed for disc diffusion. (A) 0 mM NaCl, (B) 200 mM NaCl, (C) 400 mM NaCl, and (D) 800 mM NaCl. Gentamicin discs are indicated in red, distinguishing them from nonevaluated antibiotic discs.

The influence of pre‐applied salinity stress on the antibiotic susceptibility of *E. coli* ATCC 25922 was evaluated by exposing bacterial cultures to increasing NaCl concentrations prior to the antibiogram assay. As shown in Figures 3 and 5A, early salt exposure induced a clear increase in resistance to gentamicin. The diameter of the inhibition zone decreased significantly compared with the nonstressed control (23.00 mm), reaching 17.66, 19.33, and 19.00 mm following exposure to 200, 400, and 800 mM NaCl, respectively. These findings suggest that osmotic stress encountered during the pre‐culture phase may trigger adaptive mechanisms that diminish gentamicin efficacy.

**Figure 4:**
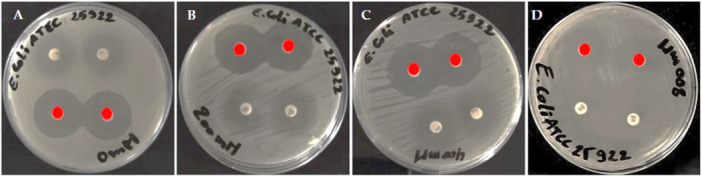
Gentamicin inhibitory activity (GN IA) against *Escherichia coli* ATCC 25922 under salinity stress applied directly to the Mueller–Hinton medium, assessed by disc diffusion. Petri dishes were incubated for 24 h at 37°C. (A) 0 mM NaCl, (B) 200 mM NaCl, (C) 400 mM NaCl, and (D) 800 mM NaCl. Gentamicin (GN) discs are marked in red, while other antibiotic discs were not analyzed in this study.

**Figure 5:**
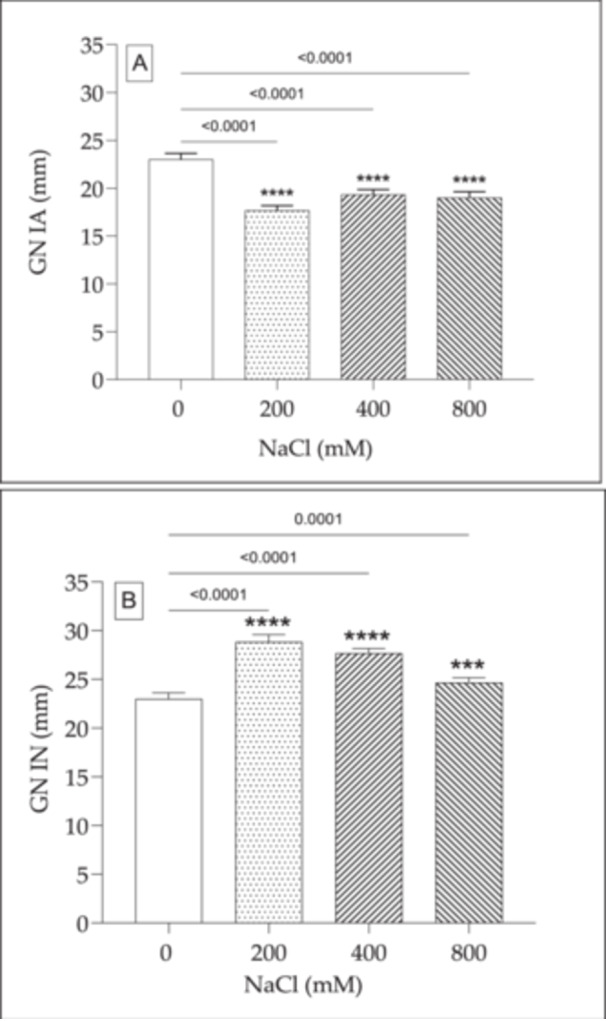
Gentamicin inhibitory activity (GN IA) against *Escherichia coli* ATCC 25922 under salinity stress conditions. (A) NaCl stress (200–800 mM) was applied to bacterial cultures prior to antibiotic testing. A concentration‐dependent reduction in the diameter of the inhibition zone was observed, indicating increased resistance. (B) NaCl was incorporated directly into the MH medium during the antibiogram. Moderate salt levels (200–400 mM) enhanced antibiotic efficacy, while high salt (800 mM) slightly increased resistance. Inhibition zones were measured after 24 h of incubation. One‐way ANOVA (Fisher LSD); ***p < 0.001, ****p < 0.0001 (*significance compared with the control condition*: 0 mM NaCl). ANOVA, analysis of variance; LSD, Least Significant Difference; MH, Mueller–Hinton.

In a separate assay, salinity stress was introduced directly into the MH agar medium used for the antibiogram. The results, presented in Figures 4 and 5B, demonstrated a different response pattern. Lower salt concentrations (200 and 400 mM NaCl) enhanced bacterial susceptibility to gentamicin, with inhibition zone diameters increasing to 29.00 and 28.00 mm, respectively. However, when the salt concentration was raised to 800 mM, a reversal in effect was observed: resistance was stimulated, and the inhibition zone diameter decreased to 24.50 mm, slightly above the baseline of 23.00 mm under unstressed conditions. These results indicate that the timing and method of salt application can distinctly modulate the antimicrobial response of *E. coli*.

##### 3.2.1.2 Antibiogram Under pH Stress

**Figure 6:**
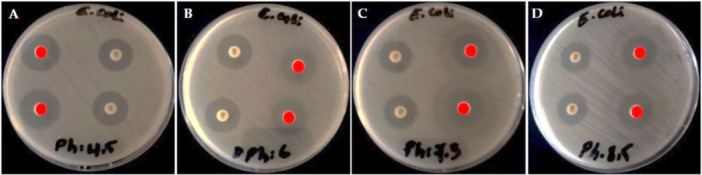
Gentamicin inhibitory activity (GN IA) against *Escherichia coli* ATCC 25922 after pre‐exposure to pH stress in Luria–Bertani (LB) broth, followed by standard disc diffusion testing on Mueller–Hinton agar medium. Cultures were incubated for 24 h at 37°C in LB adjusted to the indicated pH levels before processing. (A) pH 4.5, (B) pH 6.0, (C) pH 7.3 (control), and (D) pH 8.5. Only gentamicin discs are marked in red for clarity.

The effect of preconditioning *E. coli* ATCC 25922 cultures at varying pH levels prior to antibiogram testing was assessed to determine whether pH stress modulates gentamicin susceptibility. As shown in Figures 6 and 8A, pH pretreatment did not induce substantial changes in antibiotic sensitivity. The largest inhibition zone was observed at pH 7.3 (18.60 mm), with slight reductions at pH 6.0 (18.66 mm), pH 8.5 (16.50 mm), and pH 4.5 (17.50 mm). These modest differences suggest that transient pH stress prior to gentamicin exposure has minimal impact on the strain's resistance phenotype.

**Figure 7:**
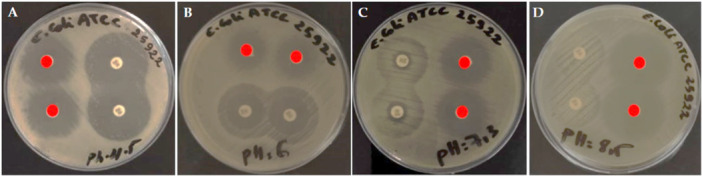
Gentamicin inhibitory activity (GN IA) against *Escherichia coli* ATCC 25922 under pH stress applied directly to the Mueller–Hinton medium, assessed by disc diffusion after 24 h incubation at 37°C. (A) pH 4.5, (B) pH 6.0, (C) pH 7.3 (control), and (D) pH 8.5. GN discs are highlighted in red. Other antibiotic discs present were not part of this analysis.

**Figure 8:**
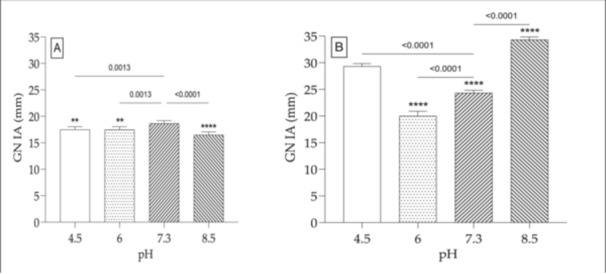
Gentamicin inhibitory activity (GN IA) against *Escherichia coli* ATCC 25922 under pH stress conditions. (A) pH stress was applied to bacterial cultures prior to antibiogram testing. Minimal changes in susceptibility were observed across tested pH values (4.5, 6.0, 7.3, and 8.5), with the largest inhibition zone at pH 7.3. (B) pH stress was directly applied to the Mueller–Hinton medium during antibiogram testing. Extremes of pH (4.5 and 8.5) enhanced antibiotic effectiveness, while near‐optimal pH conditions (6.0 and 7.3) reduced gentamicin activity. Inhibition zone diameters were measured after 24 h of incubation. One‐way ANOVA (Fisher LSD); ns, no significance; **p < 0.01, ****p < 0.0001 (*significance compared with the control condition*: pH = 7.3). ANOVA, analysis of variance; LSD, Least Significant Difference.

In contrast, direct modification of the pH in the MH agar medium during the antibiogram produced a markedly different outcome. As illustrated in Figures 7 and 8B, extreme pH values, particularly pH 4.5 and 8.5, significantly enhanced gentamicin activity, resulting in enlarged inhibition zones of 29.33 and 34.33 mm, respectively. Conversely, media adjusted to pH 6.0 and 7.3, which are closer to the strain's optimal growth conditions, reduced antibiotic efficacy, yielding inhibition zones of 20.00 and 24.50 mm, respectively. These findings indicate that environmental pH can strongly influence antibiotic performance when altered at the site of bacterial exposure.

#### 3.2.2 Case of *P. aeruginosa*

##### 3.2.2.1 Antibiogram Under Salinity Stress

**Figure 9:**
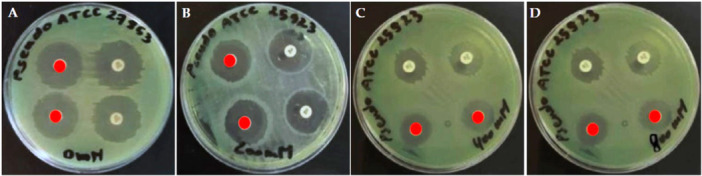
Gentamicin (GN) susceptibility of *Pseudomonas aeruginosa* ATCC 27853 after pre‐exposure to salinity stress in Luria–Bertani (LB) broth, followed by testing on standard Mueller–Hinton agar media via disc diffusion. Bacterial suspensions were prepared after 24 h incubation in salt‐adjusted LB broth. (A) 0 mM NaCl, (B) 200 mM NaCl, (C) 400 mM NaCl, and (D) 800 mM NaCl. GN discs are indicated in red for distinction.

The impact of early salinity stress on the susceptibility of *P. aeruginosa* ATCC 27853 to gentamicin was evaluated by pretreating bacterial cultures with increasing concentrations of NaCl prior to antibiotic testing. As shown in Figures 9 and 11A, salt stress induced a dose‐dependent increase in resistance to gentamicin. The inhibition zone diameter progressively decreased from 25.5 mm under nonstress conditions to 24.5, 20.5, and 18.5 mm in the presence of 200, 400, and 800 mM NaCl, respectively. These results suggest that salt preconditioning may trigger physiological adaptations that reduce gentamicin efficacy.

**Figure 10:**
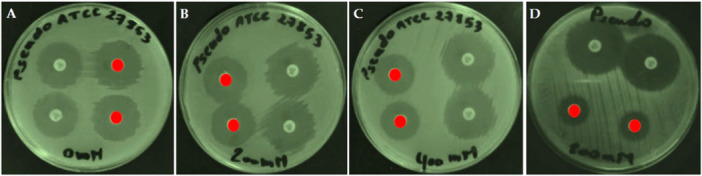
Gentamicin (GN) susceptibility of *Pseudomonas aeruginosa* ATCC 27853 under salinity stress applied directly to the Mueller–Hinton medium, evaluated by disc diffusion after 24 h incubation at 37°C. (A) 0 mM NaCl, (B) 200 mM NaCl, (C) 400 mM NaCl, and (D) 800 mM NaCl. Gentamicin discs are marked in red, as only GN was evaluated in this study.

**Figure 11:**
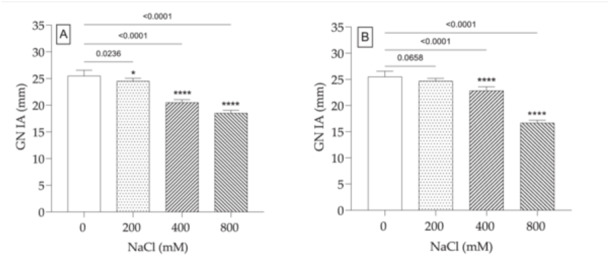
Gentamicin inhibitory activity (GN IA) against *Pseudomonas aeruginosa* ATCC 27853 under salinity stress conditions. (A) NaCl stress was applied to bacterial cultures prior to antibiogram testing. Inhibition zone diameters decreased with increasing salt concentrations, indicating enhanced resistance to gentamicin. (B) NaCl was incorporated directly into the Mueller–Hinton medium. Similar to pre‐applied stress, higher salt levels (400–800 mM) significantly reduced antibiotic efficacy. Inhibition zones were measured after 24 h of incubation. One‐way ANOVA (Fisher LSD); *p < 0.01, ****p < 0.0001 (*significance compared with the control condition*: 0 mM NaCl). ANOVA, analysis of variance; LSD, Least Significant Difference.

When NaCl was added directly to the MH medium during the antibiogram, a similar resistance trend was observed (Figures 10 and 11B). At lower salt concentrations (0 and 200 mM), the inhibition diameters remained statistically comparable, measuring 25.5 and 24.6 mm, respectively. However, higher salinity significantly compromised antibiotic effectiveness, with inhibition zones reducing to 22.8 mm at 400 mM and further to 16.6 mm at 800 mM NaCl. This consistency across both stress‐application methods highlights the significant role of osmotic conditions in modulating gentamicin activity against *P. aeruginosa*.

##### 3.2.2.2 Antibiogram Under pH Stress

**Figure 12:**
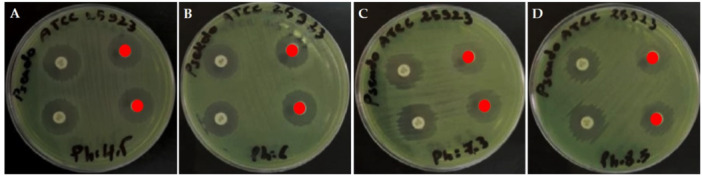
Gentamicin (GN) susceptibility of *Pseudomonas aeruginosa* ATCC 27853 after pre‐exposure to pH stress in Luria–Bertani (LB) broth, prior to disc diffusion testing on standard Mueller–Hinton medium. Cultures were grown for 24 h in pH‐adjusted LB and processed as described. (A) pH 4.5, (B) pH 6.0, (C) pH 7.3 (control), and (D) pH 8.5. GN discs are highlighted in red; other discs were excluded from analysis.

The influence of pH preconditioning on the response of *P. aeruginosa* ATCC 27853 to gentamicin was evaluated by incubating bacterial cultures at different pH levels prior to antibiogram testing. As illustrated in Figures 12 and 14A, delayed pH stress produced a comparatively modest impact on gentamicin susceptibility. At pH 4.5 and 6.0, the inhibition zone diameters were 19.5 and 19.0 mm, respectively, indicating similar degrees of antimicrobial effectiveness. Slightly reduced inhibition was also noted at pH 7.3 (17.8 mm) and 8.0 (18.3 mm), suggesting that neutral‐to‐slightly alkaline conditions may modestly impair gentamicin activity. Overall, pH‐induced stress applied prior to antibiotic exposure resulted in only limited modulation of bacterial sensitivity.

**Figure 13:**
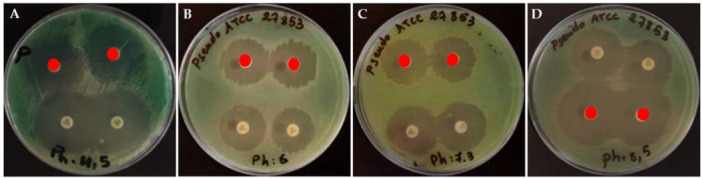
Gentamicin susceptibility of *Pseudomonas aeruginosa* ATCC 27853 under pH stress applied directly to Mueller–Hinton medium, assessed via disc diffusion after 24 h at 37°C. (A) pH 4.5, (B) pH 6.0, (C) pH 7.3 (control), and (D) pH 8.5. Only gentamicin discs (marked in red) were analyzed for inhibitory activity.

**Figure 14:**
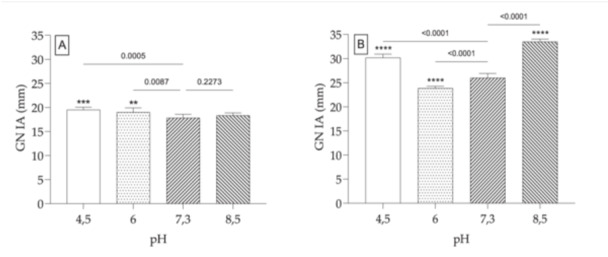
Gentamicin inhibitory activity (GN IA) against *Pseudomonas aeruginosa* ATCC 27853 under pH stress conditions. (A) pH stress was applied to bacterial cultures prior to antibiogram testing. Modest variations in inhibition zone diameters were observed across pH values 4.5–8.0, with a slight reduction in antibiotic efficacy at near‐neutral and alkaline pH. (B) pH stress was applied directly to the Mueller–Hinton medium. Gentamicin activity was significantly reduced at pH 6.0 and 7.3, while extreme pH conditions (4.5 and 8.0) enhanced bacterial susceptibility. Inhibition zones were recorded after 24 h of incubation. One‐way ANOVA (Fisher LSD); **p < 0.01, ***p < 0.001, ****p < 0.0001 (*significance compared with the control condition*: pH = 7.3). ANOVA, analysis of variance; LSD, Least Significant Difference.

By contrast, direct application of pH stress to the MH medium used during antibiotic testing elicited a more pronounced effect on gentamicin activity (Figures 13 and 14B). At near‐neutral pH values (6.0 and 7.3), the antibiotic's effectiveness was significantly reduced, with inhibition zones measuring 23.8 and 26.0 mm, respectively. In contrast, extreme pH values enhanced bacterial susceptibility: at pH 4.5 and 8.0, inhibition zones increased to 30.16 and 33.5 mm, respectively. These findings indicate that environmental pH at the site of antibiotic action can dramatically influence the efficacy of gentamicin against *P. aeruginosa*.

### 3.3 Mimicking Urinary Conditions for In Vitro Assessment of Antibiotic Response

#### 3.3.1 Physicochemical Characterization of the Urine Sample

The physicochemical properties of the collected urine sample were measured using a multiparameter probe (HANNA HI 9829). The pH of the sample was recorded at 5.68, while the EC, a surrogate for salinity, was measured at 77.6 mM. These parameters served as the basis for formulating urine‐simulated MH media.

#### 3.3.2 Pathogen Isolation and Identification

Microbiological analysis of the urine sample was performed at the Betouche Medical Analysis Laboratory (Bouira, Algeria). Using CHROMagar Orientation medium, the isolated uropathogenic strain was identified as *E. coli*, based on colony morphology and chromogenic differentiation.

#### 3.3.3 Stress Applied Directly to the Culture Medium of the Antibiogram

**Figure 15:**
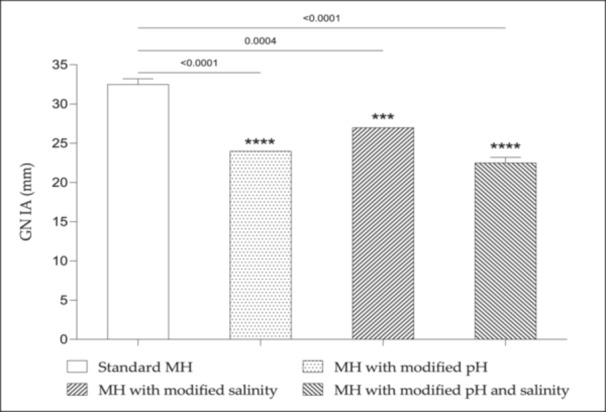
Gentamicin inhibitory activity (GN IA) against *Escherichia coli* isolated from urine, tested under standard and urine‐simulated conditions. The lowest inhibition zone was observed under simultaneous pH and salinity stress. One‐way ANOVA (Fisher LSD); *Statistical significance*: ***p < 0.001, ****p < 0.0001 (*significance compared with the control condition*: Standard MH). ANOVA, analysis of variance; LSD, Least Significant Difference; MH, Mueller–Hinton.

As illustrated in Figure 15, an initial inhibition zone of 32.5 mm was measured in the standard MH medium. However, a significant decrease in this zone was observed when the medium pH was adjusted, reducing the diameter to 24 mm. Similarly, in the medium with modified salinity, the inhibition zone also decreased, although to a lesser extent, reaching a diameter of 27 mm. Notably, an even greater reduction was recorded when both conditions, pH and salinity, were simultaneously modified, resulting in an inhibition zone of 22.5 mm.

#### 3.3.4 Effect of Salinity and pH Simulation on the MIC of Gentamicin

**Figure 16:**
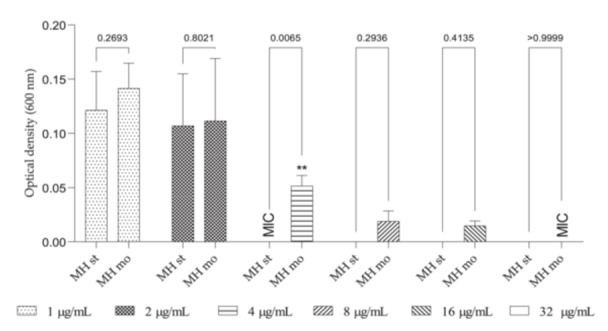
MIC of Gentamicin against the isolated *Escherichia coli* on Mullet–Hinton standard (MH st) and modified (MH mo) MH media. Statistical analysis: Two‐way ANOVA (Fisher LSD); **significant difference (*p* ≤ 0.01). ANOVA, analysis of variance; LSD, Least Significant Difference; MIC, minimum inhibitory concentration.

MIC values were determined for gentamicin in both standard (MH st) and modified (MH mo) MH broth, with the latter adjusted to match the urinary pH (5.68) and salinity (77.6 mM). As depicted in Figure 16, a clear inverse relationship was observed between gentamicin concentration and bacterial growth, as measured by optical density (OD_600_). However, MIC values varied considerably depending on the medium formulation. In standard MH broth, complete inhibition of growth was achieved at 4 μg/mL of gentamicin. In contrast, under urine‐mimicking conditions, the MIC increased eightfold to 32 μg/mL, indicating a marked reduction in antibiotic efficacy in the simulated environment.

## 4 Discussion

The bacterial strains *E. coli* ATCC 25922 and *P. aeruginosa* ATCC 27853 are routinely utilized to assess the performance of MH media in antimicrobial susceptibility assays. They are established models for evaluating the activity of a broad spectrum of antibiotics, including gentamicin, cefamandole, cephalothin, tetracycline, cephaloridine, nalidixic acid, and chloramphenicol, among others (Allocati et al. 2013; Wu et al. 2015). In this study, both strains demonstrated baseline susceptibility to gentamicin, a widely used aminoglycoside antibiotic from the deoxystreptamine subclass. These compounds, known for their potent bactericidal activity against Gram‐negative pathogens, are predominantly administered parenterally due to poor gastrointestinal absorption, with limited oral use restricted to local intestinal infections (Buxeraud and Faure 2016). Gentamicin remains a critical option in treating serious infections; however, its efficacy can be significantly influenced by environmental factors that mimic clinical or extra‐clinical niches, such as those found in human urine.

Our findings reveal that exposure to simulated urinary conditions, particularly variations in pH and salinity, markedly altered the antibiotic susceptibility profile of *E. coli*. These changes were most evident in the inhibition zone diameters and the MIC, with the latter increasing from 4 µg/mL in standard MH broth to 32 µg/mL in modified media adjusted to urinary pH (5.68) and EC (77.6 mM). This eightfold increase strongly suggests that urine‐like abiotic stresses attenuate the effectiveness of gentamicin.

Exposure of bacterial cells to high‐osmolarity conditions reduces water activity within the cytoplasm, which in turn disrupts the function of proteins and other macromolecules. This stress response results in the inhibition of several essential biological processes, including macromolecular synthesis and DNA replication (Csonka 1989). Similarly, pH also plays a critical role in influencing microbial structure and function. Microorganisms, including bacteria, are particularly sensitive to the hydrogen‐ion concentration in their environment. Variations in pH can significantly affect the structure and activity of large proteins, such as enzymes. Alterations in pH may lead to protein denaturation and changes in the ionic charges of these macromolecules, thereby impairing their biological functions (Ratzke and Gore 2018).

Bacterial adaptation to environmental stresses is mediated by global regulatory mechanisms, such as the RpoS sigma factor, which plays a central role in stress responses and stationary‐phase survival in *E. coli* (Battesti et al. 2011). RpoS expression increases under nutrient limitation and osmotic stress, promoting cellular resilience (Dong and Schellhorn 2010). Salt stress in particular affects protein synthesis, membrane fluidity, and intracellular solute concentrations, thereby influencing both growth and antibiotic susceptibility (Y. J. Zhang et al. 2019; Imhoff 1986).

Our results align with earlier findings suggesting that the osmotolerance of *E. coli* is limited under high salinity conditions, with growth reductions exceeding 80% under NaCl concentrations of 800 mM (Ghoul et al. 1989). This can be attributed to disruptions in turgor pressure and the need for active osmoprotective mechanisms. These include the uptake of potassium ions, synthesis of compatible solutes such as glutamate, and, under more severe osmotic conditions, the accumulation of betaine via the ProU transport system (Lucht and Bremer 1994). The energetic cost of these adaptive mechanisms may impair growth and alter membrane permeability, reducing the influx of antibiotics, such as gentamicin.

To date, relatively few studies have investigated the behavior of antibiotics under conditions where bacteria are subjected to environmental stress. Such stress not only affects the bacterial cells themselves but may also influence the structure and efficacy of the antibiotics. Consequently, the interaction between bacteria and antibiotics under these conditions is inherently unstable. The findings of this study clearly suggest that several factors critically influence the outcome of antibiotic treatment, including the dosage, the environmental origin of the bacterial strain, the antibiotic's mechanism of action, and the physicochemical conditions under which the bacteria–antibiotic encounter occurs.

The observed decrease in gentamicin activity under high salinity is consistent with the findings of Coronado et al. (1995), who demonstrated that osmotic stress can reduce antibiotic efficacy through multiple mechanisms. These include (1) chelation of antibiotic molecules by inorganic ions, (2) conformational changes that impair antibiotic penetration, and (3) altered membrane permeability, which collectively reduce intracellular drug accumulation.

Similarly, acidic stress alters bacterial morphology, surface charge, and membrane dynamics (Bearson et al. 1997; Guan and Liu 2020). Acid‐induced modifications of the cell envelope can compromise interactions between antibiotics and porin channels, particularly for cationic compounds, such as aminoglycosides. These effects may be driven by hydrogen‐ion competition for membrane‐binding sites, as described by Abraham and Duthie (1946) and more recently elaborated by Aranda‐Díaz et al. (2020). The net result is reduced antibiotic uptake and decreased efficacy under acidic conditions. Although our findings are consistent with stress‐adaptation mechanisms reported in the literature, the underlying molecular mechanisms were not investigated in this study. Therefore, the proposed roles of RpoS, membrane remodeling, osmoprotective responses, and altered gentamicin uptake remain biologically plausible hypotheses rather than experimentally demonstrated mechanisms. Future studies should combine molecular analyses with biofilm models to validate these mechanisms and determine whether urine‐mimicking conditions similarly influence gentamicin susceptibility in biofilm‐grown bacteria, thereby extending the clinical relevance of the present findings (Thakur and Kumar 2026).

Furthermore, structural instability of aminoglycosides under low pH may contribute to their inactivation. Wiegand et al. (2015) reported that acidic environments can degrade the trisaccharide backbone of gentamicin, thereby diminishing its antibacterial activity. The dual stress of low pH and high salinity, therefore, likely exerts a synergistic effect, reducing gentamicin stability and bacterial permeability simultaneously, thus suggesting the substantial MIC elevation observed in urine‐mimetic conditions.

One limitation of this study is that modifying MH agar by changing its pH and NaCl concentration may affect antibiotic diffusion and other physicochemical properties of the medium, thereby influencing inhibition zone diameters independently of bacterial physiology (Washington et al. 1978). To address this limitation, we complemented the disc diffusion assays with broth MIC determinations under the same urine‐mimicking conditions. The eightfold increase in gentamicin MIC supports that the reduced antibiotic activity cannot be explained solely by altered diffusion.

Collectively, these findings underscore the importance of simulating host‐relevant microenvironments when evaluating antimicrobial activity, especially in cases of therapeutic failure based on a standard antibiogram. The physiological conditions of urine, such as its moderately acidic pH and variable ionic strength, can modulate both pathogen behavior and antibiotic pharmacodynamics. Incorporating such parameters into susceptibility testing may yield more clinically relevant data, particularly for UTIs caused by opportunistic pathogens, such as *E. coli*.

## 5 Conclusion

This study suggests that antibiotic efficacy and bacterial phenotypes are strongly influenced by environmental conditions rather than being fixed properties. Standardized antibiogram protocols, while essential for routine clinical practice, may not fully reflect the physicochemical conditions encountered at the site of infection and, consequently, may not always predict antimicrobial performance under physiological conditions. Our findings provide initial evidence that incorporating infection‐site physicochemical parameters into AST may improve the interpretation of gentamicin susceptibility results, particularly in UTIs.

The present work also highlights the potential value of environment‐specific susceptibility testing frameworks that better capture in vivo variability. However, as this study focused exclusively on gentamicin, the applicability of these findings to other antimicrobial classes remains to be established. Future investigations should therefore evaluate whether similar environmental modulation occurs with other antibiotics commonly used for UTIs, including fluoroquinolones, β‐lactams, nitrofurantoin, and fosfomycin, as well as across a broader range of clinically characterized pathogens. Such studies may contribute to the development of more physiologically relevant antibiogram protocols, particularly when standard susceptibility testing fails to predict therapeutic outcomes, ultimately supporting more personalized and clinically relevant antimicrobial therapy. Finally, Future studies using transcriptomic, proteomic, and functional approaches will be needed to validate the molecular mechanisms underlying the observed changes in gentamicin susceptibility.

## Author Contributions

**Abdelwahab Rai:** conceptualization, methodology, software, investigation, resources, data curation, writing – original draft. **Mahdia El‐djohra El‐amina Saoud:** conceptualization, methodology, software, resources, data curation, writing – original draft. **Nesrine Tabchouche:** validation, formal analysis. **Hamdi Bendif:** funding acquisition, project administration. **Stefania Garzoli:** supervision, writing – review and editing. **Lotfi Mouni:** visualization, writing – review and editing.

## Funding

The authors have nothing to report.

## Ethics Statement

The study was conducted as part of a master's research project in the Department of Biology at the University of Bouira, Algeria. The clinical *E. coli* isolate used in this study originated from a single anonymized urine specimen collected during routine clinical laboratory practice. The research was restricted exclusively to laboratory analyses of the bacterial isolate, including microbiological characterization and AST. No human participants were recruited or enrolled in the study, no intervention was performed on any individual, and no identifiable personal, demographic, or clinical information was accessed, collected, stored, or analyzed by the investigators.

According to the research policies currently applied by the Department of Biology, University of Bouira, laboratory research involving only anonymized bacterial isolates recovered from routine clinical specimens, without the recruitment of human participants or the use of identifiable personal or clinical information, is generally not considered to require prior review or approval by an Institutional Ethics Committee. An official institutional statement issued by the Department of Biology, University of Bouira, and signed by the Deputy Head of the Department in Charge of Scientific Research, has been provided to the Editorial Office to further clarify the institutional framework applicable to this study.

## Conflicts of Interest

The authors declare no conflicts of interest.

## Data Availability

The data that support the findings of this study are available from the corresponding author upon reasonable request. The data are not publicly available due to privacy or ethical restrictions.
